# Observations on Cancrum Oris, as It Appeared in the Cincinnati Orphan Asylum, from February, 1842, to May, 1847

**Published:** 1847-08

**Authors:** Thomas Carroll

**Affiliations:** Cincinnati, Ohio


### WESTERN JOURNAL

OF

### MEDICINE AND SURGERY.

AUGUST, 1847.

ART. I.—*Observations on Cancrum Oris, as it appeared in the Cincinnati Orphan Asylum,* from February, 1842, to May, 1847. By THOMAS CARROLL, M.D., of Cincinnati.

Cancrum oris has probably existed from time immemorial; we, however, can only trace its history to the times of Van Swieten, it being first noticed by him, since which period authors have occasionally mentioned it; but special attention has only been paid to the matter by writers within the last few years. From these we learn that it has mostly been found in low, damp, or marshy situations, and where children have been much crowded together, as in the various charities reared expressly for the reception of orphans, or in the very densely populated parts of cities. It has also been observed that the badly fed, the badly clothed, and the emaciated, from whatever cause, are but too often obnoxious to its ravages It seems, too, that children under the age of ten years have been almost alone subject to its attacks. It has been observed as far north as Sweden, and in northern Germany, Great Britain, etc., but never, so far as I know, within the tropics.

No season of the year is exempt from its visitations, though its fatality is no doubt greater in warm than in cold weather, and in the former it prevails more extensively than in the latter. Indeed, when it does exist during cold weather, it often has had its origin during the preceding summer or autumn. But of all the causes which produce this horrible disease, none has hitherto been so prolific in its production, either in Europe or this country, as the crowding together children of a tender age in large numbers, as in asylums or hospitals. Indeed, so far as my observation has extended in the investigation of this disease as it has appeared at the Cincinnati Asylum, I am impelled to the belief that the most devoted attention, the best directed arrangements, the most wholesome food and the most comfortable clothing will not altogether prevent the disease in institutions of the kind, particularly among children under four years of age.

According to my apprehension, the leading predisposing causes in this climate are malarious in character, for I have never seen the affection in localities where remitting and intermitting fevers did not exist. Should this prove to be a truth, an important avenue to relief will be found, as it would then be obvious that immediate removal into an elevated and nonmalarious locality would be among the best means of cure, and should always be the first resorted to.

But as it is my only object to speak of cancrum oris as it has been presented to me at the Cincinnati Orphan Asylum, I shall mostly confine myself to this object, unless it be by way of illustration. I shall therefore begin with a description of the house and its locality, with observations on its malarious situation and some other causes that have led to the disease.

The establishment is situated in the northern part of the city, on the east side of the Miami canal, and within a few hundred feet of it. It is in an open space of considerable extent, and from this fact it would appear that the diseases incident to the city population would have but little influence in producing corresponding affections, particularly those growing out of the crowded and too filthy condition of some parts of the city.

The house contains about twenty-five apartments, which are mostly large and well constructed for ventilation; those in the basement are in some respects an exception, but dampness is more injurious in them than anything else. In this story alone are the kitchen and dining-room, with other apartments not designed for the use of the children, and therefore can have but little influence on their health.

The dormitories, with the exception of that belonging to the nursery, are situated in the third and fourth stories. So soon as the children leave these rooms in the morning, they are opened and continue so throughout the day; but at night they are either partially or altogether closed, according to the condition of the weather; and the rays of the sun are prevented from entering the apartments by Venetian blinds. In the nursery and dormitory connected with it these regulations also prevail. This dormitory is large and would hold many beds; there are, however, in the general not more than fifteen, which are single and are kept in the best possible order. The children when they dress in the morning are taken into the nursery, and then the windows of the room are opened, and ventilation is continued throughout the day. The children are not allowed to return until they retire for the night. Both these rooms are heated by stoves, and occasionally by heated air. They are kept without carpets, to avoid the accumulation of animal excretions. Carpets were used, but soon after my appointment I discovered that much filth necessarily accumulated in them, which could not be prevented by any means short of removal. And further to secure these rooms against fetid or hospital smell, the chloride of lime in solution or the fluid chloride of soda properly diluted was used occasionally during several summers with a happy effect. The latter seemed to answer a better purpose than the former.

Thus none of the rooms have been occupied more than twelve hours out of the twenty-four, and ventilation and cleanliness have been particularly attended to, with a single exception, which is the sick-room. That has hitherto been almost constantly used from week to week for the same melancholy purpose, and in not a few cases, I think, with a most unhappy effect. That the sick should be removed from one ward to another every twelve hours, I have no doubt; this regulation would allow a complete ventilation of the domicils of the sick, and would confer on them what is always essential to health, pure air. It has long been my own habit when sick to use two rooms, removing from one to the other every few hours; and nothing has seemed to tend more to comfort than this, and in a few instances appeared to lay the foundation of recovery.

With regard to this matter, I fear there is not a hospital in the world which is rightly managed; for I believe that almost everywhere the unfortunate inmates spend day after day in the same wards, and but too often die in the contaminated air which has been for months or at least weeks mixed with animal effluvia.

It may here be proper to observe that the diet of the inmates has always consisted of mixed kinds. Those over the age of two or three years are allowed animal food at least once a day, such as beef, mutton, etc. Milk and various kinds of vegetables are also admitted, with molasses, weak coffee, etc. In the nursery, milk and bread, sago, arrow-root, with, occasionally, soup, are mostly used.

From the foregoing it might be inferred that but little room for the vitiation or impoverishment of the fluids could at any time have existed, and therefore that little could result from this cause calculated to establish that peculiar irritation which terminates in cancrum oris. Yet we find that it is more frequent here than in the city or neighborhood. This frequency must in some degree be attributed to the fact, that children are often admitted who have been badly taken care of, badly clothed, and badly fed. Management of this kind not only leads to debility, but is the nursery of various species of cutaneous diseases and bowel affections, which not unfrequently are the indirect cause of ulceration of the mouth. The children of vitiated parents are occasionally brought into the house about the time of the death of one or both parents, or sometimes these diseased persons throw their offspring on the world within a few hours, weeks or months after birth. Of course they soon linger and die, and sometimes become affected with gangrene of the mouth before death. The children, too, of the consumptive or of those laboring under general scrofula very often find their way into this establishment.

Again, children are admitted from different parts of the world, and that, too, before any acclimation could have taken place. The foreign emigrant reaches our city in poverty and laboring under disease; he dies and leaves his children to the charity of strangers. But not only the foreign emigrant, but the American who has left the home of his fathers in the east, and has fixed his residence somewhere in the malarious regions of the west, is overcome by the hardships and unhealthfulness of his situation; he dies, and his children, by the charity of a friend, find their way back thus far towards their father-land. These not unfrequently reach the asylum in the most wretched condition, both as regards health and clothing.

Those children who have been admitted from the malarious situations of the southwestern States are peculiarly obnoxious to cancrum oris. Indeed, that peculiar species of anemia which those regions produce in children of a tender age renders them peculiarly susceptible of the disease under consideration. But it is not uncommon to find ulceration of the mouth at the time of admission, especially in those laboring under diarrhea, which will be further noticed by and by.

Out of more than a hundred annual admissions, there must of course be many admitted laboring under chronic diseases.

The cancrum oris appeared early in the asylum, but its first serious visitation, I think, took place in 1837, in conjunction with measles or immediately following it. The number of deaths resulting from the disease at that time I cannot state, but have understood that the mortality was severe. At the time of my appointment in 1842, there were but thirty-eight inmates in the house; about ten of these were under three years of age, and of course were in the nursery. The number, however, before the end of the year rose to sixty, and since January, 1843, there have been between sixty-five and one hundred.

During the years 1842 and 1843 there were twenty cases of cancrum oris, of which two proved fatal. All or nearly all of them labored under chronic diarrhea during the period of sloughing; indeed, the two cases which proved fatal had chronic diarrhea for many weeks before the gangrenous condition of their mouths supervened, and only seemed to succumb to the general consequences of that disease. Although there was sloughing of the cheeks internally and of the gums, yet the cheeks were not penetrated by the mortification at death.

This affection has not proved fatal in a single case in which it has appeared as an idiopathic disease; it might therefore be claimed as only fatal when caused by other and more formidable affections. This indeed appears to me to be the way in which it must be viewed in coming time by the medical philosopher, particularly when it assumes its graver aspects.

In the Cincinnati Asylum its grave character was mostly felt when following measles or some other cutaneous disease; then danger was to be apprehended, and especially when connected with measles.

In some instances it followed remitting and intermitting fever. A case of this kind occurred in 1844: A boy five years of age had remitting fever, and was convalescent on the 1st of April; but on the 24th, some small ulcers appeared in the mouth, and the gums were inflamed and dark colored, with slight ulceration. The patient, however, recovered in a short time by the usual treatment. Aside from this case, two others came down from 1843, both of whom recovered. No other cases occurred during 1844, and it may be stated that unusually good health prevailed throughout the year, there being but three or four fatal cases in the house.

To convey a just idea of the influence of eruptive diseases in the production of cancrum oris, I shall now give a short history of them as they have appeared in the house during the time under consideration. The varioloid appeared in December, 1843, and continued until some time in January, 1844. Out of thirty-eight patients, which constituted the whole number, all recovered, and none of them were marked; but what was strange to me was, that this affection left no disposition to cancrum oris in any case.

During 1845 there was more disease of various kinds in the house, and more cases of cancrum oris. The varicella made its appearance on the 15th of January in a single case, and in four others on the 18th. After this a number of cases took place and interlocked with the measles, which was manifested on the 21st of February, and continued in the house until May. Ten fatal cases occurred during this time. Immediately preceding the occurrence of the measles, the whooping-cough had existed and had produced an unfavorable state of the system in some subjects for the reception of the measles, and appeared to be the indirect cause of death in a few instances.

On the first appearance of the measles, the patients seemed to do well; but during the month of March and part of April the weather was cold and dry, the atmosphere having that kind of constitution which is generally complained of during that season of the year. Probably this condition had a tendency to make the measles, during the time of its recession, have an unfavorable influence on the mucous surface of the alimentary canal; for in most of the cases it became much irritated, congested, and, in the lower bowels, intensely inflamed; for although diarrhea first supervened, dysentery soon followed and became unmanageable in nearly all the fatal cases. With these symptoms were soon united loss of appetite, quick pulse, and inflammation of the mucous membrane of the mouth. This latter symptom was accompanied by swelling of the lower part of the face. Had the weather been as warm as it was when the measles visited the house in 1846, some of the patients would no doubt have had sloughing of the mouth; but the coolness of the atmosphere appeared to prevent that excessive inflammation which terminated in sloughing during 1846; while the mucous membrane of the large intestines suffered a most intense inflammation, which would probably not have been the case had the weather been milder. Although six of the fatal cases succumbed directly to the measles, yet it was my opinion at the time that all these would die during the then coming summer. Three of them were laboring under chronic diarrhea, and the other three had congenital syphilis, which was rapidly destroying its victims. As has been observed, these six patients all took the measles, but not until they had been some time in the house, for it seemed that a great concentration of the poison was necessary before they could be brought under its influence. The mouth of one of the three last named became badly ulcerated before death.

From May until June no epidemic skin disease occurred; there was, however, in July one fatal case of cancrum oris, and one other that recovered. During the autumn of this year five cases of cancrum oris occurred, though of rather a slight character. These cases improved under treatment, but three of them remained in a slightly diseased state until the invasion of the measles some time after.

On the 24th of December, 1845, scarlatina made its appearance in a child ten years of age, but a month elapsed before another case supervened; and from that time until the 25th of February, twenty-five cases occurred; of this number but one died. In conjunction with scarlatina, the varicella existed in a few cases. The foregoing shows the amount of eruptive disease previous to the month of March, and demonstrates how little the varicella and scarlatina did in the way of producing sloughing of the mouth. This further shows that in the asylum measles has been the only epidemic which has obviously caused cancrum oris.

On the 24th of March, twenty days after the last case of scarlatina, a child about four years of age was found to have cancrum oris. There was a slough on the inside of the lower lip near its junction with the jaw, and the glands in the neighborhood were swollen and tender to the touch. There were also two other slight cases which entered the following month, showing, as has been observed, that there were two cases less than in December, 1845; these three, however, were of the five of that month. One had recovered before May, but was retaken, and in the other two the ulceration had been continued in consequence of chronic diarrhea. These cases eventually proved fatal because of the supervention of the measles, which broke out in the house on the 14th of May.

On the 10th of May a child was admitted, who on the 14th sickened; and on the 21st another inmate had catarrhal symptoms, which resulted in the eruptive stage of measles on the 24th. After these, thirty-five other cases made their appearance before the middle of July. In 1845 there were seventeen more cases, yet the mortality was less than in the following year.

During the months of May, June and July, there were seventeen cases of cancrum oris. Three of them came down from the preceding months, and four of them still existed in August. Out of these, ten succumbed, all of which had the rubeola either before the sloughing began or during its progress.

Before entering on the symptomatology of this affection, I shall make a few further observations on those circumstances which render individuals liable to its attacks. In the first place, all those who are anemic are obnoxious to the affection, and are especially so when diarrhea is present either in its acute or chronic form. In the second place, those who are in a state of hyperæmia not unfrequently take the disease. This class comprehends all the robust who are great eaters, and whose muscular action is slow, and who possess most of the other characteristics of the phlegmatic temperament. In this species of constitution skin diseases of a chronic character are apt to exist, though it may be said with equal truth that those temperaments bordering on the sanguineous are almost as subject to those affections; and probably in this mixed temperament cancrum oris appears as often as in the former. And in the third place, as has been noticed, those who have been subjected to malarious and damp situations, or who have been enveloped much in filth, are more subject to the disease than those who have come into the house under opposite circumstances. But in the fourth place, that class who have by nature a tendency to scrofula, or who have it in a state of development, are apt to have the disease, and, above all other classes, are most likely to die. Of this more will be seen by and by.

In giving the symptoms of this affection, I shall simply describe what I have observed, as I have noticed no description which meets exactly what I have seen. In most instances there has been either constipation or diarrhea to a greater or less extent, and the alvine evacuations have been unusually offensive and not of proper color or consistence. There has often been fullness of the abdomen, and in the more serious cases there has been sallowness and emaciation, with sometimes a bloated condition of the face and extremities even before any tendency to sloughing of the mouth had been observed; yet those of a full habit, as has been noticed, under certain circumstances have been subjected to it, though to a much less severe degree.

It may be best for the sake of perspicuity to divide the disease into acute and chronic, though there is not in fact much foundation for such a classification. It is true, however, that the disease runs its course in a few days in not a few instances, while at other times it continues for weeks or even months. This latter occurs in those cases which might be considered idiopathic.

In the chronic form, the first local symptom is generally observed in the gums, in either the upper or lower alveolar process, and on only one side—it being a pretty general law, that it is found only on one side, though, as will be hereafter seen, there are deviations from this apparent law. The affected gums are mostly connected with but two teeth, and are of a dark red color, with occasionally a white band round the tooth, which soon appears started from it; this increases for a short time, when it ulcerates and is still further separated from the neck of the tooth. The inflammation travels toward the connection of the lip or cheek or gums, but does not, as might be expected, travel up the mucous surface of the cheek, but stops short. An ash-colored spot now makes its appearance a little back of the angle of the mouth and in the centre of the mucous surface. This spot is often half an inch in length, and the width is generally about half the length. To this there are exceptions, as it is not unfrequent for two or three round ash-colored spots to be scattered along the mucous surface from the angle of the mouth back; sometimes there is but one of these. A dark red-colored areola surrounds each of these spots, which are in an inactive or congestive state. When one of these ash-colored spots is felt, it is found to have a hardened base which extends beyond its edges, or, in other words, is larger than it is. This solidified spot often involves the cheek but partially, but in some cases it penetrates through the lip or cheek, and is found on the outer side, solid and smooth at first, but after a little there appears a red spot on the skin. When this involution of the skin takes place, the most serious consequences may be anticipated. After it has arrived at this point, and even before, the face swells considerably, particularly on the side affected. Now the hardening with redness gradually extends, and the slough internally has gradually extended, and instead of keeping on a level with the surrounding surface as hitherto, it drops below, becomes darker and more rigid; and the tendency to gangrene of the cheek is evinced by increased hardness and extending solidification. The inflamed spot on the denuded surface may be confined within a narrow space, or may extend over a considerable portion of the cheek; either in general proves equally fatal. But to return: The mortification of the cheek or lip, when once begun, preceded by the swelling and inflammation, spreads—if in the lip, in all directions, but if in the cheek, more rapidly upwards. The angle of the mouth or edge of the lip is not for some time involved. It would seem that the vitality of the lip longer resists the morbid action than the surrounding parts.

After the solidification has passed through the cheek, which may take place within the space of a few days or be extended to a week or more, its inflamed centre becomes a dark spot, which within twenty-four hours after is a third of an inch or more in diameter. After twenty-four hours more, it has doubled its size, and its surface now sinks below the surrounding parts, and appears over the surface dry, and does not seem large enough to fill the space, but is slightly disposed to separate from the still living tissue. The mortification continues to extend, and generally seems to delight in the more fleshy parts of the cheek. The patient now has increased thirst, which has hitherto been great. He often heaves deep sighs, and has increased frequency of breathing, with considerable fever during the after part of the day, and occasionally has slight chills in the morning. At night, and indeed during the day, he tosses himself from side to side, and has delirium; this latter symptom is not very often present during the day.

He now cannot be prevailed upon to open his mouth, and carefully avoids stretching the narrow slip of lip through which the mortification has not yet penetrated, but swallows as though there was no danger. The lip, however, gives way, and the most frightful aspect of the disease is now unveiled. The child still talks, and, if several years old, speaks with composure of its approaching end, and when not delirious is pathetic and resigned. After the patient has arrived at this point, he may survive some days, or, indeed, it is possible for him to recover, though this seldom happens.

But we must return and trace other symptoms within the mouth, etc., for the disease does not always appear in the garb above described. It sometimes first shows itself in the gums of the upper molar teeth, and not unfrequently in those of all the upper teeth between the molares, as I have sometimes observed in the gums of a single cuspidatus or bicuspidatus, and spread from thence. Occasionally the gums of the upper or lower incisor teeth become first involved; and in a few instances I have seen those of both the lower and upper simultaneously affected, but only in malignant cases following measles. Three of the latter character took place in July, 1846; in these diarrhea supervened at the close of the eruptive stage of rubeola; their appetite was lost during the measles, and did not return; not even the slightest amount of nourishment could be borne, and there was constant thirst without the power of retaining drink; the surfaces were cold, or rather below the natural warmth; while the pulse was in power too weak, and too frequent.

When I first examined the mouths of these patients, I found the gums had an ash-colored appearance; the incisor teeth were even now loose, and within twenty-four hours they began to drop out. One of these patients Prof. Harrison saw with me in consultation; the gums were then swollen, and of a slightly venous color. Eighteen hours afterwards, when I again saw the patient, the gums had separated from the teeth and the teeth were loose; in thirty-six or forty-eight hours more the patient succumbed. When this child took the measles, it was in bad health and its constitution was of the most delicate kind. Its parents had resided somewhere in the State of Mississippi, and had long labored under malarious influences; the children being born in that country and under those influences, were of course delicate and were remarkably slender. One other of this family fell a victim to this disease.

This sudden separation of the gums from the teeth brings us to another condition of this affection, which is twofold in its character, and widely different from that which alone affects the soft parts:

Where a confined or quite limited ash-colored slough forms around the root of one or more teeth and penetrates to the alveola, and the teeth become loose, a different set of symptoms make their appearance, and are, in most of the cases which occur, added to those which have been described. In acute cases, often in a day or two, the teeth become loose and are easily plucked out by the fingers; but in the less acute forms several days may elapse before this will take place. After the teeth have come out, the alveolar process comes to view, and is of a dark brown or black color. After the loss of the teeth, the gums soon become more healthy in appearance; indeed, I have seen them in at least two instances become quite healthy looking. From this it would seem that their separation from the periosteum had relieved them from disease for the time being, and would go to prove that the affection had begun in the immediate covering of the bone. But this diseased condition of the periosteum and bony tissue is not wholly confined to the alveolar process; it spreads more widely, runs up the bones of the face, reaches the orbit, and dips into one or both antra, and in some cases attacks the bones of the nose, causing a most horrible ozæna. This latter symptom, as will be seen, may last for many weeks, and the patient eventually recover, though, if in the vomer, with a deformed nose.

To give the reader a more accurate idea of this malady in cases where the periosteum and bony tissue are diseased, I shall report the following case, and shall relate another when I come to the treatment:

A girl ten years old had been admitted, with three others of the same family, some weeks before the 30th of May, 1846. She had fair complexion and light hair, and was tall and slender, with apparently feeble muscular power. On the 30th of May she took diarrhea, for which was prescribed one-twelfth of a grain of calomel and half a grain of Dover's powder, with five grains of prepared chalk, every eight hours. This did not check the diarrhea, and on the 1st of June she evinced symptoms of measles. Her eyes were much inflamed, which was soon after followed by the eruption of the measles, which went through their regular stages, notwithstanding the unabated continuance of the diarrhea. To relieve this, I directed Hope's mixture in teaspoonful doses every few hours. During the continuance of the measles, severe cough with bronchitis was present, though not in an alarming degree; but on the 9th these symptoms were worse, and there was added severe pain in the chest, and with the expectoration were mingled large quantities of blood. As the pain was in the right side, a blister was ordered on that part, and antimonials were given as freely as the stomach and bowels would bear. On the 10th she was much relieved of pain, and the bloody expectoration was diminished; she, however, continued to cough; the skin was now cool and the pulse more regular. During this time the diarrhea remained unabated, or at least had not subsided; Hope's mixture was continued. As the cough still existed, on the 11th ten drops of laudanum were ordered with the other medicine, by which she was much relieved through the night. On the 12th she had improved much, but was unfortunately directed to take one or two small portions of brandy, on account of debility; this rather invited a return of fever than relieved the weakness; the cough seemed to increase, and the diarrhea remained unsubdued, though not severe.

On the 15th, about fifteen days from the first appearance of the measles, the gums began to ulcerate or slough around the last incisor of the right side, the ulceration running back until the gums of the two adjoining teeth became involved. The hæmoptoe still continued on the 16th and 17th, with evidently undiminished congestion of the right lung; but on the 18th there was no bloody appearance. On this day the sloughing had extended to the gums of all the upper incisor teeth, and the bone had become bare for some distance up, involving the whole depth of the alveolar process. The solid nitrate of silver was applied to the sloughing parts, with a wash composed of water and the muriated tincture of iron, which in my absence was prescribed by Dr. Conner. Under this treatment the soft parts much improved, and eventually looked healthy, but the affection of the periosteum still increased in diseased appearance; all the incisor teeth had become loose, and were soon picked out by the patient.

At this stage of the disease the lips were intensely red, and the upper one was swollen. The weather was warm, the mercury being up to 90°, which condition seemed much to enfeeble the patient. Her pulse had all the time been over

100 per minute, but now beat 140 or more, and was more feeble; during the after part of the day she had more fever, and at night was slightly delirious.

From this period until the 28th there was no increased sloughing of the soft parts, except where they were in connection with the periosteum; there they were loose and gangrenous, and the bone had become much more denuded. The right side of the face had much increased in swelling, so that on the 29th the right eye was nearly closed, and the upper lip on this side was solidified, with but little redness; the left side of the face participated slightly in the swelling. Mortification began on the 1st of July in the hardened lip, and by the evening of the 2d it had considerably extended. The emaciation by this time had become very great, the pulse was much sunk, and there was constant restlessness. She died on the 3d. At the time of her death the whole of the upper alveolar processes had become black as far back as the last molares; all of the teeth but these had come out. The sloughing and periosteal inflammation had run round the last molar tooth.

About eighteen hours after death I sawed out the upper alveolar processes. The saw passed through the antra, which were found filled with a black and semifluid matter, the result of gangrenous action; this was particularly the case in the right one, the other being affected in its osseous lining in a much slighter degree; the former was black, the latter brown. On the right side the soft parts were separated from the bones to the edge of the orbit, but on the left the separation had not extended more than half way. The cancellated substance of the jaw was filled with dark matter, or had a dark color when cut through; this diseased condition was much less than that of the external covering.

But we have still to notice the pathological condition of the mucous surface and of the glands of the mouth. The latter in some instances secreted largely, while the former only occasionally secreted more than usual if as much. Indeed, more danger may generally be apprehended where the mouth is dry than in its opposite condition. There are occasionally about the onset of the affection small ash-colored spots on the point of the tongue, and on one or both edges; these spots are tender, and the tongue swollen, especially in their immediate neighborhood. This state of the mouth does not, however, belong to chronic cases accompanied with diarrhea, or where there is much debility, but rather to those in which there is plethora or hyperæmia, and where the affection is of an acute character.

In one instance I observed one-half of the roof of the mouth swollen and inflamed, which latter became more intense as it ran back, and only terminated when it reached the fauces. In this case the inflammation terminated by resolution.

The glands of the fauces with the submaxillaries occasionally became involved in this sloughing gangrene, without either the gums or lips being affected; yet there can be very little doubt of a close connection if not a positive identity of the two diseases. The following case will go to show how this affection operates when located in the fauces and glands:

A boy twelve years of age, a son of Dr. Belknap, now of Iowa, was taken with the measles some time in June, 1846. He got through them pretty well, but imprudently exposed himself to rain just at the close of the recession, and was taken with remitting fever. He lay in the fever two weeks, and suffered much with pain in the head, delirium, etc. The treatment was to some extent mercurial, with warm sponging, etc. When he began to recover he was quite feeble, but soon sat up, took food pretty well, and it was hoped that a perfect convalescence would ensue; this, however, did not take place. Within a few days he began to complain of his fauces, and had a hacking cough; the tonsils swelled, as well as the surrounding cellular membrane and the glands in the immediate neighborhood. In a few days abscesses formed in the tonsils and in the cellular tissue in the neighborhood, which now burst one after another, and discharged a most fetid matter, thick and dark-colored; some of the mouths of these abscesses could be seen, and had a ragged aspect. The patient emaciated rapidly, the skin became dry, and the cough was troublesome. Under these circumstances he died within a few days after the bursting of the first abscess.

Twenty-four hours after death I examined the body in the presence of Dr. Dodge, of this city, and the father. We found all the soft parts of the fauces in a gangrenous state, even the root of the tongue being involved in the mortification, and the mucous membrane covering the glottis and epiglottis, as well as that dipping into the larynx, thickened and dark. The lungs, which some had thought deeply implicated, were found healthy, without even an evidence of congestion.

Professor Drake saw another case with me in the same family, which it was feared might result in the same manner as the first; but there were no abscesses formed, nor was there any sloughing. Prof. D. gave it as his opinion, that in cases of mortification of the mouth following measles there was a close relation with epidemic erysipelas of the face, etc.

It may not here be improper to make a few observations on the difference of diagnosis between cancrum oris and that sloughing affection which sometimes follows the use of mercury. Although there has not been a case in the asylum since I have attended it which obviously grew out of the improper administration of this mineral, yet I have at different times seen this mercurial disease, and have examined it with some care. In a majority of those cases a marked difference existed between them and cancrum oris, at least so far as regarded the diseased condition of the osseous tissue. In the latter it has been seen that the periosteum became affected before the substance of the bone, and was not stationary, but ran like the continuous inflammation of the mucous tissues, only with less rapidity; but in the former the substance of the bones suffered more intensely than their immediate covering, although they both seemed to be implicated.

I have now before me a specimen which I took a few years ago from a living individual of this city, which consists of the two anterior molar teeth of the right side of the under jaw. The cancellated portion of the bone in this specimen appears to have been more deeply affected than the harder parts or the periosteum. The teeth are now only slightly loose in the bone, but on the inside the cancellated structure is much destroyed; yet the part approaching the alveoli is the most perfect, and there still remains a shell covering the fangs of the teeth, which, however, is not perfect. The dens sapientiæ had made its way to the surface of the alveolar process, but the bony covering on the side adjoining the tooth came out and left it with a healthy appearance, and it continued in the jaw afterwards.

On the external side of the specimen the gums adhered at the time of its removal, though the adhesion was not perfect. The line of separation of the part removed was bounded by the base of the antrum which was connected with the part removed. The bicuspidal tooth of that side came out, as more than half of its socket had been absorbed.

In this case suppuration had existed for some months before the bone and teeth were removed. Salivation had been produced eight months before, but the patient had only been confined for the last four months, during which time the pus found an outlet around the loosened bone; the discharge was profuse and caused much debility. Within a few hours after the removal of the bone, the patient, who was sixteen years of age, was relieved of all pain; his recovery was rapid, so that within about three weeks afterwards he was cured.

About the year 1830 I was consulted with regard to two other cases which followed scarlatina, but which were obviously the result of large doses of calomel administered not only during the disease, but afterwards. These cases took very nearly the same course as the one just related: the molar teeth of the under jaw, with the alveolar process connected with them, came out, and I believe that in at least one of the cases those of both sides were removed, but not until after the lapse of several months. Now, if these cases had resulted from cancrum oris, the periosteal covering would first have been inflamed, the teeth would have been picked out within a comparatively short period, and the bone would have been entirely denuded of soft parts, and black. Such was not the result in any of these cases.

Where mercury commits ravages on the soft parts, both sides are usually if not always affected equally, and internally there is little difference in either side, as the inflammation of the mucous membrane is pretty equally diffused, though I believe it is not unusual for one side to be a little more affected than the other. In cancrum oris the matter is very different: here the inflammation is but seldom equally diffused over the surface of the mouth, and the face suffers on but one side, and often in but one lip. Instead of all the gums being inflamed, those of but a few teeth are affected, and the color is darker at the outset. These, indeed, seem to be important facts in the diagnosis, for it scarcely ever happens that all the gums even of one jaw slough from this affection.

In two cases of sloughing of the face from the improper use of mercury which have come under my observation, both cheeks sloughed at the same time, which was to me an evidence that mercury had been the cause. This, however, needs further observation before it could with safety be relied on as a rule.

*Treatment.*—Observation soon led me to the conclusion that in cancrum oris the mucous surface of the whole alimentary canal was implicated. There was either diarrhea, as has been observed, or constipation, or at least a vitiated condition of the alvine evacuations, for they were very offensive in nearly every case, and were not of a proper color or consistence. Of course the treatment must necessarily be governed by the condition of the general system. In the chronic forms of this disease we will separately notice that which occurs in the plethoric first, and then that which is connected with emaciation, etc.

Where, in these plethoric cases, there was fullness and tenderness of the abdomen, and offensive evacuations with slight disposition to constipation, and also where the mouth was rather disposed to run than to be dry, my course was to give an emetic either of the tartrate of antimony or of ipecacuanha. A teaspoonful of antimonial wine was given about every thirty minutes until emesis resulted; when ipecacuanha was directed, ten grains were given and repeated, but when this did not produce vomiting, no more was administered. I then let the medicine act as a cathartic, which it generally did within six, twelve or twenty-four hours. In cases where it failed, I directed one grain of calomel with ten or twelve of jalap to the larger patients, and less to the smaller ones. This was repeated every few hours until pretty free evacuations were brought about. After the first evacuations, oil or magnesia was occasionally given; calomel and jalap were, however, mostly depended on in plethoric patients, and I think generally did better than any other cathartic.

The treatment of the mouth next demanded attention, and this at first was much more mild than after I had had some experience. I began by using a gargle of three grains of nitrate of silver to an ounce of distilled water, with which the mouth was carefully washed every four or six hours. I soon discovered, however, that this solution was too weak, and that one with ten grains to the ounce answered a much better purpose. Rubbing was not allowed in the application of any wash. Once or twice a day was considered sufficient for the last named solution to be used.

But I found that where slough was present, with considerable inactivity of the connecting vital parts, a still more active application was needed; I therefore applied solid nitrate of silver around the slough, which seldom failed to excite a more healthy action. The mere application of the caustic to the surface of the dead parts could of course be of no use; but when they were broken up by a pencil of the nitrate, and it was brought directly in contact with the living base, it must necessarily have an effect for good or evil. There is no doubt in my mind that it was beneficial in a very large proportion of cases. The pain produced by the application lasted but a few moments, and was succeeded by a more agreable sensation than had been felt previously. Experience taught that generally the caustic could not be beneficially applied oftener than once in forty-eight or seventy-two hours, and that sometimes even longer intervals were necessary. Usually within twenty-four hours after the application, the zone surrounding the slough assumed a more healthy appearance, and not unfrequently the slough dropped off or disappeared before the end of the third day. Between the applications of the solid nitrate the solution before mentioned was used once a day or even less often. This was done to prevent those unhealthy congestions which preceded sloughing or ulceration.

When the gums were in an ulcerated or sloughing state, the solid nitrate was run over them until they bled slightly. This generally did well, but a pretty strong solution was usually sufficient. But for this affection of the gums, as well as for the sloughing of other parts, another remedy had a great influence and is now a favorite application in the house; this is the fluid chloride of soda. I at first directed it with eight additions of water, but for some time past have used only three or four parts of water. It was then applied every six or eight hours over every part of the surface of the mouth, but latterly it has been used more frequently. In the very worst forms of the disease it at once destroyed the fetor, and, except in the incurable cases following measles, had a happy effect in disposing the affection itself to disappear. Indeed, in a few recent cases which have appeared in the house, no other local application has been used.

Other remedies were tried at various times, but mostly when there was little or no chance of anything being useful. It has been stated that Dr. Conner prescribed the muriated tincture of iron in one case, and that it had no useful effect; but it will have been seen that no means could have been of use. Further trials must decide the benefit that may be derived from this article, and I think it will be found useful in ulceration of the mouth even where there is sloughing. Dr. Dodge recommended diluted nitrous acid; but I did not adopt his idea of the matter, because I preferred the action of nitrate of silver. This preference was founded upon the fact that the animal fluids have less power in neutralizing the action of nitrous acid than that of nitrate of silver, and that therefore the caustic power of the former might prove injurious to parts possessing little vital action. Prof. Harrison called my attention to another prescription—sulphate of copper, Peruvian bark and alcohol; this I used in the case which the professor saw, but with no good effect. I must acknowledge, however, that the case was a very bad one. The gums were only moderately swollen when Dr. H. saw it, but in eighteen hours they had separated from the teeth, which were loose and soon came out; and the patient did not live more than two days after the doctor made his visit. Prof. Drake informed me that he had seen cancrum oris cured by a solution of carbonate of potassa; but I have no experience myself, and of course cannot decide as to the propriety of its use. I, however, many years since adopted this medicine as the very best in malignant sore throat, and in all cases of ulceration of the tonsils it was long my practice to prescribe it in weak solution; but a gargle of the nitrate of silver has nearly superseded all others in my more recent practice. Yet I am not certain but that I was as fortunate with the former as with the latter remedy. But after all, the nitrate of silver, either in substance or solution, stands first in my estimation as a local remedy, and next to it the fluid chloride of soda, and then the various astringents, either vegetable or mineral. The latter should be used with moderation, taking care that a few hours should elapse between the applications.

We must, however, return to the point where we left off the consideration of the constitutional treatment, and further pursue the steps that have been taken in the asylum. When patients were laboring under the chronic form of the disease, connected with emaciation and debility, I have found nothing equal to sulphate of quinine as a tonic; it has proved particularly beneficial in cases where regular or irregular chills were present, followed by fever. There is now, while I am writing, a case in the asylum that will serve to show the influence which this medicine occasionally has:

A male child three and a half years old was brought into the house three months ago, and at that time was emaciated almost to the last degree. Fever came on every afternoon, preceded by cold fits or chills; the skin for many days was dry, and the pulse feeble and frequent; the alvine evacuations were too light colored and offensive; much of the hair seemed to have been broken off near the scalp, and the scattered remnant had lost its original pliability and softness. Under these circumstances it became necessary to administer not only tonics, but alteratives; I therefore directed two grains of rhubarb with one-twelfth of a grain of calomel to be given every night for a few times. At the outset, however, ten grains of ipecacuanha were given, which acted as an emetic; this has been repeated five times. Quinine was very soon directed, and early gave evidence of doing great good in the case. Half a grain dose was given three times a day for some time, with the happiest effects: the skin became softer, the chills soon disappeared, the appetite returned, the emaciation began to disappear, and the hair resumed its healthy appearance. During this time, warm salt baths were used once a day. Under this course the child has gradually improved.

About the time that he seemed to be rapidly improving, I found that the gums of the upper incisor teeth had slightly separated from them and were ulcerated. This I had for some weeks anticipated, and had therefore given but little mercury, choosing rather to excite the liver by emetics. But as the quinine had been laid aside for some time, I now resumed its use in half grain doses, but oftener than before. Under this course the child soon improved, and now, although there is still some ulceration, he is running about, and weighs several pounds more than he did six weeks since. Fluid chloride of soda diluted has alone been used as a local application, and it has seemed to do well in producing a healthy action of the gums.

It has not been my object to give large doses of quinine in chronic cases of the kind we have been considering, because time is allowed for a gradual removal of the affection. Large doses not unfreqently disagree with the stomach, and when disorder is once induced it is difficult to restore the organ to its wonted powers. I therefore have mostly prescribed from an eighth to half a grain every four, six or eight hours to children two or three years of age, which has usually been continued during several weeks. Where much debility has existed, small quantities of wine or brandy have been directed, and frequently with good effect, particularly when given with quinine, as then it often caused this medicine to be more easily retained.

During the continuance of the above course, I have in all cases where the strength of the patient would bear it, directed the cold shower-bath or sponging every morning; for the feeble the warm salt dash has been directed once a day or less, according to circumstances. The application of cold water I think has in many instances been of singular service, and should be considered indispensable where the patient appears more lively than usual in a short time after it.

In a few cases which occurred during 1844 and 1845 I tried the long continued use of iodine, on the principles laid down by Lugol. In several patients the general health was improved, the appetite benefited, and the flesh increased; but I was unable to discover any direct influence on the sloughing process. It must, however, from its tendency to invigorate the system, at least in occasional cases, render the gangrene less ready to increase or progress towards a fatal result; and with the aid of proper local applications it will therefore be useful in those cases where weeks must elapse before a cure can be effected.

I shall report two cases in which Fowler's solution was administered with advantage, though the reader may very readily conclude, from the time employed in doing it, that the medicine but slowly caused the disease to retreat:

Two brothers, one eight years old and the other six, from one of the malarious districts of Illinois, were admitted during the autumn of 1843. Both were laboring under cancrum oris, were somewhat emaciated, and withal had a bloated appearance. Each took an emetic of ipecac. and was purged; one drop of Fowler's solution was given three times a day; and the slough and adjoining parts were touched with a solution of nitrate of silver. As their mouths did not improve very rapidly, the arsenic was continued during three months without seeming to have produced its specific effect. Both cases now resulted in a cure.

But on the 8th of March, 1844, both had that kind of fever which so often appears about this time of the year in persons who have been subjected to malaria during the preceding autumn, or who have had fever from their influence. Each was directed to take an emetic with cathartics. The elder got better, but the younger one had an immediate return of ulceration of the mouth. The ulceration extended into the fauces, and the parotid glands swelled considerably but did not suppurate. I now directed one grain of calomel with ten grains of jalap, which acted pretty freely as a cathartic; after this action had ceased I directed two drops of Fowler's solution three times a day. The local application was simply the nitrate of silver in solution. On the 28th of the month, about twenty days after the relapse, the condition of the ulcers had much improved, and all the sloughing had taken place. The arsenic was steadily given for two weeks, and the gargle of nitrate of silver was continued until the 17th of April, when the patient was discharged cured.

In these cases the arsenic seemed to act as a slow and powerful tonic, even without its specific effect being produced—a result that I have often observed on other occasions. Indeed, the most happy effects may be obtained by the use of this medicine without its being given to that extent which is necessary to produce dropsical effusions.

In those forms of cutaneous disease named porrigo, eczema, lichen, ecthyma, and those which come under the appellation of squamæ, more particularly when united with cancrum oris in its chronic form, should generally be treated with arsenic in the way above indicated. This course will mostly do better than any other, and would, if early resorted to, often prevent cancrum oris. Ecthyma, which has been in the house for several months, was treated by various external applications with some of the common alteratives, but I was unable to remove it except by the arsenical solution, which has now nearly eradicated it.

This would probably be the right place to make some observations on the proper diet in chronic cases; but I shall first consider the acute form of the disease, and then make some statements upon this subject.

Acute cancrum oris is that which generally follows eruptive diseases, or sometimes accompanies them. The chronic form not unfrequently becomes acute upon the invasion of measles or some other dermal affection, though this last condition may rather be considered a chronic affection suddenly rendered worse by conjunction with an eruptive disease. This affection, when it becomes endemic or epidemic with measles, has proved the most intractable of all the diseases we have had to contend with. Indeed, it seems to me that when it is thus connected, it will prove a most mortal disease, and will probably even bid defiance in a majority of instances to every effort of the healing art, especially when it occurs during warm or hot weather. Medicine has seemed to have but little influence in arresting its progress, and one symptom has crowded upon another toward a fatal termination, despite every effort to the contrary.

It is true, that in all instances irritation of the bowels with diarrhea was present, and this irritation frequently resulted in inflammation at least of the mucous surface of the colon. These symptoms were the leading and probably the real cause of death, since, when these affections followed or accompanied measles in the spring of 1845, death resulted in a number of cases where there was no sloughing of the gums or any part of the buccal cavity. At that time there was more intense inflammation of the mucous surface of the bowels than in 1846. The mouth affection then appeared to be but one symptom of the disease, the immediate cause being an unhealthy condition of the fluids. Even the bones owned the deteriorated state of the fluids by the inflammation of their periosteal tissues, as well as by the morbid condition of their cancellated structure, as has already been seen; this condition, however, is found only in the bones of the face and head. It has already been observed that the acute form of cancrum oris began, with few exceptions, on the decline of the eruptive stage of measles. This sloughing condition of the mouth in all the cases connected with measles was preceded by diarrhea, and, in those which were fatal, continued to their close.

As the congested state of the mucous surface of the mouth seemed to participate in the irritation of the bowels, even if it were not caused by it, I directed early efforts to the subjugation of this affection. The sudden stoppage of the diarrhea was not attempted by astringents, as it was thought that harm must result instead of good. But when this affection could be arrested by the establishment of healthy excretions and secretions at an early period, the cases generally or always did well.

It is obvious to the reader, no doubt, that mercurials could be of little use in diarrhea following measles, where there was already much debility, and where no difficulty seemed to exist in the liver more than in the other abdominal organs. The trials that I made with hydrargyrum cum creta did not fulfil my expectations; indeed, I became satisfied that it was of no use; and as acidity did not seem to be present to much extent, prepared chalk did not have much effect. As there was certainty of inflammation both of the upper and lower bowels in a majority of cases, it became important to arrest it if possible; but here little could be done, for the patients could not with safety bear the application of leeches. In one case, however, a single leech was applied to the abdomen of a child two or three years of age, early in the summer of 1845, by the advice of Dr. White, of this city. Before the application there was tenderness with some fullness of the abdomen. The tenderness was somewhat relieved and the child appeared considerally better for a few days, but it succumbed after two or three weeks. In this case there was no sloughing of the mouth, but merely a slow inflammation.

Dr. White also directed my attention to the use of Hope's dysenteric mixture, as all the diarrheal cases of 1845 had dysenteric symptoms at the same time. Since that period I have very freqently used this mixture in very nearly the proportions laid down by Dr. Hope: I direct laudanum ʒss, or sometimes f. ℈ij, with f. ʒj of nitrous acid, all added to ℥viij of camphorated water. To a child two or three years of age I have ordered about a teaspoonful every six hours, altering the quantity according to the age and other circumstances of the patient. No medicine has been more useful than this in cases of diarrhea where there has been much debility with ulceration of the gums; it has generally lessened the number of evacuations within twenty-four hours in manageable cases, and in the incurable has at least relieved pain. In these inflammatory cases warm alcohol was mostly directed as an external application, but other substances were occasionally used, such as several kinds of spices combined with flour and whisky or vinegar and water. In addition to Hope's mixture, brandy or wine was directed in a number of the cases that occurred during the summer of 1846.

In those cases where the diarrhea was very profuse, sugar of lead appeared to be more beneficial than any other remedy of the astringent kind, as it seemed almost certain that it would not increase the inflammation. I accordingly prescribed it in a few cases during 1845 and 1846, but derived no benefit in any case. In one the diarrhea suddenly stopped, and the abdomen swelled very much; the patient died under these circumstances, and I therefore quit the use of the lead in cancrum oris with diarrhea.

In the acute condition of this affection, no use could be made of ipecac. in many of the subjects of it, though in a few, where there was but little debility, advantage was derived from its use. The following case will show that it may be used with advantage in the acute form, though this case was not connected with measles:

A child three years of age had apparently a good constitution, and, before the attack of cancrum oris, had evinced no evidence of bad health; but in June, 1846, a slough suddenly formed on the inner side of the upper lip, very near the left angle of the mouth. When the patient came under my notice, the gangrene had penetrated half way through the lip and was half an inch in diameter. I immediately applied solid nitrate of silver, taking care to make it reach the living substance beneath the slough; it was also applied to the surrounding mucous surface, so that a more active circulation might ensue. An emetic of ipecac. was then administered, followed by ten grains of jalap and one grain of calomel, which acted freely as a cathartic. Half a grain of quinine was now given every four hours for some time. At the end of three days the mortified parts dropped out, contrary to my expectations, and the cavity soon filled up with healthy granulations. Good health was rëestablished in ten days after the attack.

I now anticipated that if this child took the measles, which was then in the house, cancrum oris would almost certainly return, and the patient eventually fall a victim to it; but in this I was disappointed, for the child did take rubeola, went through it well, and had no return of the previous disease.

The favorable result of this case further demonstrates the advantage of vomiting and purging in all cases where the patient can bear them without producing much prostration.

The following instance will show how low a patient may become with cancrum oris and yet be susceptible of measles, and how certainly death follows such attacks. In this case the child would no doubt have succumbed even if it had not taken rubeola, yet a number of cases would have resulted favorably but for the joint influence of the two affections.

A boy aged four years was seized with diarrhea about the middle of May, 1846. On the 23d a slough had formed in the mucous membrane opposite the molar teeth of the right side, and on the 29th had begun around the superior incisors. Diarrhea still continued; to relieve this, I directed ten grains of ipecac. to be given, which did not produce emesis, but acted as a cathartic. Hope's mixture was also directed, and by the 1st of June the diarrhea had much abated. The patient now took a grain of quinine every six hours for a number of days, and the bowels were kept sufficiently in check with Hope's mixture.

On the 3d and 4th the incisor teeth came out, the slough still spreading; the upper alveolar process was nearly black and bare throughout more than half of its extent. On the 6th there was no improvement of the periosteal appearances, and the soft parts had become worse. During the next three days there was no appreciable increase of the sloughing, but the inflammation of the periosteum had increased or spread farther. During this time the appetite continued good, or rather became better, which accounts for the stationary condition of the sloughing from the 6th to the 9th.

On the 10th he was doing well. On the 11th, the bone was still more bare; otherwise the mouth was more favorable. On the 15th the sloughing was worse, and the pulse too high and frequent; this seemed a little strange, as I had expected its force to be diminished; it was explained, however, by the appearance of the eruptive stage of measles next day, The patient survived only until the 18th.

The following case will show how measles altered the condition of cases which would in all probability have recovered if they had been suffered to run their course without the superaddition of this disease:

A girl aged eight years had symptoms of cancrum oris on the 1st of May, 1846. The disease gradually got worse until the 31st, when an ash-colored pedicle appeared on the mucous surface of the mouth, just over the masseter muscle. To relieve this, nitrate of silver was freely applied. Quinine had been given and was continued in half grain doses every few hours. On the 3d of June the slough did not look quite as well; the caustic was therefore again used, and the quinine was continued for some time, with a repetition of the nitrate of silver every other day. This course was pursued until the 9th with a happy effect, as the mouth had much improved,

But on the 11th measles began to be manifested, and on the 14th the eruption was well out and of a good color. The caustic was now used with caution, and quinine entirely laid aside. The infusion of serpentaria was directed in order to keep out the cruption—a medicine which has had a happy effect in congestive measles. The other cheek now became affected and was solidified, and I feared that the slough which was forming on the inner side would penetrate the cheek. By the 17th the measles had disappeared, and the mouth was no worse. On the 18th the caustic was used, and on the 19th the mouth had slightly improved. The bowels, hitherto disordered, were now in a healthy state. During the 20th, 21st and 22d the appetite was good, but the ulcer was deeper in the cheek.

I concluded to try iodine, and prescribed it; but it soon disagreed with the stomach, or rather irritated the bowels. In giving this medicine after the disease had become so active, an error was committed, and I can scarcely tell in what way I came to make the prescription. Chloride of soda was directed, and had its usual and desired effect of destroying the fetor. Notwithstanding all that was done, the gangrene penetrated the lower lip on the right side. Before this took place, the cheek and lip swelled very much, and the lip assumed the dusky red color always preceding mortification. The patient survived until the 29th, and death did not take place until the right side of the upper lip had become involved in the mortification. During the illness of this patient the appetite in the general was good, and this decided me in the opinion for a long time that she would recover; but when measles supervened, I gave up the case as lost.

The following case will further show the influence that rubeola had in conducing to a fatal termination in cancrum oris, and will also demonstrate how this affection may be unfavorably influenced by cutaneous diseases of a chronic character:

N. Fisher, aged about four years, had porrigo up to the 5th of June, 1846, when it nearly disappeared. On the 10th the eruptive stage of measles was manifested, the eruption being then of a good color; but on the 12th it became purple or nearly black. This disappeared, however, on the use of serpentaria, etc. No other unfavorable symptom occurred until the 28th, when sudden sloughing of the gums of the upper incisor process began most rapidly.

On the 29th sloughing of the gums of both the superior and inferior incisor teeth took place, with inflammation of the periosteum of the alveolar processes connected with those teeth. The gums were of course almost immediately separated from the teeth and processes, while the teeth became loose and began to drop out before the 2d of July, when the patient died.

I might cite other cases to prove that measles, taking hold of children who have recently been or are still laboring under chronic skin diseases, is apt to result unfavorably; but this would now be improper, except so far as these affections are connected with the disease under consideration; and in this experience has demonstrated that it is more apt, under these circumstances, to result in death, than where but one dermal affection exists during the invasion of cancrum oris.

It remains to consider the possibility of cure when the periosteum or bones are involved in this affection. I have seen no case recover where the alveolar process became denuded, and the periosteum black or dark colored. In all instances the periosteum took on a continuous inflammation, which extended so far that the soft parts eventually sloughed, and death soon followed. I treated a case, however, following measles, in 1846, of the character just spoken of, except that instead of attacking the alveoli, the bones of the nose became at least partially carious. The child, about five years old, was seized with rubeola, which went regularly through all its usual stages. Diarrhea supervened, however, towards the close of the eruptive recession, and the mucous surface of the mouth gave evidence of congestion and slight inflammation. But it was in the nasal organs that disease was seriously developed. The Schneiderian membrane began to inflame, and discharged much fetid pus having the appearance of diseased bones. The ozæna continued for weeks, the patient meantime laboring under slight diarrhea and rapid emaciation.

This case was treated with moderate but long continued doses of sulphate of quinine, and six drops of diluted fluid chloride of soda three or four times a day. Dover's powder and chalk were also occasionally given, but Hope's mixture was chiefly relied on to control the diarrhea.

After the general debility had partially subsided, taking the patient to the country frequently had the happiest effects. But although the general health constantly improved after the first month, the nose continued to discharge the same kind of unhealthy matter; so that within eight weeks after the attack of measles, the septum narium sank considerably, leaving the bridge of the nose without support below the ossa nasi, and caused some deformity. The child continued to improve notwithstanding this deformity, and was in good health in less than four months after the invasion of rubeola.

Were it necessary, I could cite another case at length, farther proving the point in question; but this is unnecessary. It may be proper to observe, however, that the periosteum of a limited portion of the occipital bone became carious after the close of the measles, and a morbid abscess formed, which, on being opened, discharged considerable unhealthy pus. The membranes of the brain became affected, a single convulsion supervened, and death immediately followed.

In taking a retrospective view of what has been said, it may not be improper to observe, that the mortality of May, June, and July, of 1846, was apparently much increased during those months by the intense heat of the weather, the two latter in particular. I frequently visited the house early in the day, with the hope of finding the little sufferers refreshed by the night, but was nearly always disappointed, and found, to my mortification, that they had farther traversed the thorny way of pain and weakness. During these months several rooms were occupied by the sick, which was of great moment, as at one time we had nineteen patients in bed, and for many days the number ranged between ten and twenty. Of this number a considerable proportion, as has been said, had cancrum oris, a majority of which cases proved fatal, and in most of them no remedial agent had any good effect. Like the cholera or some of the forms of scarlatina, it bade defiance to our *art.*

The diet of the patients, when under treatment and during convalescence, has been of as substantial a kind as could be admitted; both animal and vegetable food have entered into it. In the acute forms of the disease, however, but few of the patients took any food, and often what was taken was rejected. Indeed, this inability to take food was one of the most alarming symptoms of the disease, either before or during the time of sloughing. On the contrary, when food could be taken with ease and relish, a lively hope might be indulged of eventual recovery. In the acute form, rice, sago, arrow-root and boiled bread and milk constituted almost the only food; sometimes a little soup was given in these cases. In the milder forms of the disease, animal food, with bread and milk and sometimes vegetables, was used in such quantities as could be easily borne.

In conclusion, it may not be improper to say that there has been but little tendency to cancrum oris for a number of months past. To what to attribute this I am at a loss. Possibly this malady may nearly disappear, and may again return, owing to the constitutional condition of the atmosphere. Its banishment may possibly be owing to a change in the culinary operations; indeed, the bread has been much better of late than formerly, and it is possibly true that other kinds of cookery have also been better, but this is only conjecture. I am not satisfied, however, that sour bread, which formerly was occasionally used, is injurious to even the health of children; yet I fear that it is not so favorable to digestion nor yet to the proper condition of the fluids as that which is destitute of acid fermentation.

Should the managers of the asylum construct rooms so that the children of the nursery can be removed every twelve hours from one to another, for the purpose of ventilation, etc. I should hope for much in the improvement of the health of the younger inmates.

May, 1847.

